# Antiprotozoal Activity Profiling of Approved Drugs: A Starting Point toward Drug Repositioning

**DOI:** 10.1371/journal.pone.0135556

**Published:** 2015-08-13

**Authors:** Marcel Kaiser, Pascal Mäser, Leela Pavan Tadoori, Jean-Robert Ioset, Reto Brun

**Affiliations:** 1 Parasite Chemotherapy, Swiss Tropical and Public Health Institute, Basel, Switzerland; 2 University of Basel, Basel, Switzerland; 3 Drugs for Neglected Diseases *initiative*, Geneva, Switzerland; Johns Hopkins Bloomberg School of Public Health, UNITED STATES

## Abstract

Neglected tropical diseases cause significant morbidity and mortality and are a source of poverty in endemic countries. Only a few drugs are available to treat diseases such as leishmaniasis, Chagas’ disease, human African trypanosomiasis and malaria. Since drug development is lengthy and expensive, a drug repurposing strategy offers an attractive fast-track approach to speed up the process. A set of 100 registered drugs with drug repositioning potential for neglected diseases was assembled and tested *in vitro* against four protozoan parasites associated with the aforementioned diseases. Several drugs and drug classes showed *in vitro* activity in those screening assays. The results are critically reviewed and discussed in the perspective of a follow-up drug repositioning strategy where R&D has to be addressed with limited resources.

## Introduction

Neglected tropical diseases (NTDs) such as leishmaniasis, human African trypanosomiasis and Chagas’ disease affect the poorest people in developing countries. NTDs are responsible for substantial global morbidity, mortality and economic losses [1]. Leishmaniasis is endemic in 88 countries around the globe with 350 million people living at risk and there are an estimated 1.5 to 2 million new cases per year [2]. Human African trypanosomiasis is transmitted by tsetse flies and the disease threatens millions of people in over 20 countries in sub-Saharan Africa. Due to reinforced surveillance and vector control, the number of reported cases has come down from approximately 40,000 to less than 8,000 cases in the last 15 years [3]. Chagas’ disease is endemic in 18 countries in Central and South America. It is estimated that 120 million people are at risk of infection and that 8 million are already infected [4]. Malaria, caused by *Plasmodium spp*., is one of the most devastating diseases in developing countries, with 200 million reported cases in 2013, causing 584,000 deaths in that year [5]. There are only a few drugs available for the treatment of these diseases. These drugs have associated liabilities including lack of efficacy, severity of side effects, high costs or lack of practicality for field use, all of which constitute hurdles in terms of access to treatments for patients. To combat these neglected tropical diseases, new and better drugs are needed. The next generation of drugs needs to be effective and safe, orally-available, and with a long shelf-life in tropical field conditions. These drugs should form the basis for simple, short-course drug administration regimens (maximum 10 days, ideally 1–3 days for malaria) amenable for use in drug combinations, to prevent the emergence of resistance. The latter demand applies to all diseases but is especially important for malaria due to the global spread of drug resistance to existing antimalarials including artemisinin-based derivatives, for which the first cases of delayed clinical efficacy have already been reported [6].

There are several strategies to develop new drugs against NTDs. *De novo* drug discovery and drug development is a highly rational approach but it is a lengthy and expensive process [7, 8]. Alternatively, a drug repurposing strategy can be used as a fast-track approach guided by established Target Product Profiles (TPP) [9, 10], but this can only be considered with drugs which are active *in vitro* in relevant assays. Existing drugs or drug- like molecules are ideal to start with, because these molecules often have known pharmacokinetics, safety profile and are approved by the regulatory authorities [11, 12]. When a new application has been identified, the molecules can be rapidly advanced into clinical trials. Here, we report the *in vitro* activity against *Trypanosoma brucei rhodesiense*, *Leishmania donovani*, *Trypanosoma cruzi* and *Plasmodium falciparum* of 100 registered drugs selected for their potential to be repurposed for antiprotozoal diseases based on their respective TPPs.

## Methods

### Chemicals

Antiviral compounds were received from the NIH AIDS Reagent Program (USA). Other compounds were purchased from Sigma-Aldrich.

### Bioassays

The *in vitro* activities against the protozoan parasites *T*. *b*. *rhodesiense*, *T*. *cruzi*, *L*. *donovani* axenic amastigotes, *P*. *falciparum*, and cytotoxicity assessment against L6 cells were determined in a serial drug dilution assay (100–0.002 μg/ml) as reported in Orhan et al 2010 [13]. Drug stock solutions (10mg/ml) were in DMSO (100%). The maximal DMSO concentration in the assays was 0.5%, which does not influence parasite growth. Negative controls did not contain DMSO. Selectivity index (SI) was calculated as IC_50_ L6 cells/IC_50_ parasite.

#### Activity against *Leishmania donovani* intracellular amastigotes assay

Mouse peritoneal macrophages (4 x 10^4^ in 100 μl RPMI 1640 medium containing 10% heat-inactivated FBS) were seeded into wells of Lab-tek 16-chamber slides. After 24 h 1.2 x 10^5^ amastigote *L*. *donovani* in 100 μl were added. The amastigotes were taken from an axenic culture grown at pH 5.4. Four hours later, the medium containing free amastigote forms was removed and replaced by fresh medium. The next day the medium was replaced by medium with or without a serial drug dilution of seven 3-fold dilution steps covering a range from 30 to 0.04 μg/ml. Parasite growth in the presence of the drug was compared to control wells. After 96 h of incubation, the medium was removed and the slides fixed with methanol for 10 min followed by staining with a 10% Giemsa solution. Infected and non-infected macrophages were counted for the control cultures and those exposed to the serial drug dilutions. The infection rates were determined. The results were expressed as a percentage reduction in parasite burden compared to control wells, and the IC_50_ was calculated by linear regression analysis. The collection of mouse peritoneal macrophages was done at the Swiss Tropical and Public Health Institute (Basel) according to the rules and regulations for the protection of animal rights ("Tierschutzverordnung") of the Swiss "Bundesamt für Veterinärwesen". The animal work was approved by the veterinary office of Canton Basel-Stadt, Switzerland (permission number 2374).

#### In vitro cytotoxicity with mouse peritoneal macrophages

Mouse peritoneal macrophages were seeded in 96-well microtitre plates at 10^4^ cells/well in 100 μl RPMI 1640 medium containing 10% FBS and 2 mM l-glutamine. After 48 h 100 μl fresh medium was added with or without a serial drug dilution of seven 3-fold dilution steps covering a range from 100 to 0.14 μg/ml. After 96 h of incubation, the plates were inspected under an inverted microscope to assure sterility. Alamar Blue (20 μl of a solution consisting of 12.5 mg resazurin (Sigma) dissolved in 100 ml phosphate buffered saline) was added to each well and the plates incubated for a further 4 h. The plates were then read with a Spectramax Gemini XS microplate fluorometer (Molecular Devices Cooperation, Sunnyvale, CA, USA) using an excitation wave-length of 536 nm and an emission wavelength of 588 nm. The IC_50_ values were calculated by linear regression from the sigmoidal dose inhibition curves using SoftmaxPro software (Molecular Devices Cooperation, Sunnyvale, CA, USA). Podophyllotoxin was used as control.

#### *T*.*b*.*rhodesiense* (STIB900) acute mouse model

The STIB900 acute mouse model mimics the first stage of the disease. Four female NMRI mice were used per experimental group. Each mouse was inoculated i.p. with 10^4^ bloodstream forms of STIB900, respectively. Heparinized blood from a donor mouse with approximately 5x10^6^ /ml parasitaemia was suspended in PSG to obtain a trypanosome suspension of 1×10^5^ /ml. Each mouse was injected with 0.25 ml. Compounds were formulated in 100% DMSO, diluted 10-fold in distilled water. Compound treatment was initiated 3 days post-infection on four consecutive days for all administration routes (i.p., p.o.) in a volume of 0.1 ml/10 g. Three mice served as infected-untreated controls. They are not injected with the vehicle alone since we have established in our labs that these vehicles do not affect parasitaemia nor the mice. Parasitaemia was monitored using smears of tail-snip blood twice a week after treatment for two weeks followed by once a week until 60 days post-infection. Mice are considered cured when there is no parasitaemia relapse detected in the tail blood over the 60-day observation period. Mean relapse days were determined as day of relapse post-infection of mice. In vivo efficacy studies in mice were conducted at the Swiss Tropical and Public Health Institute (Basel) according to the rules and regulations for the protection of animal rights ("Tierschutzverordnung") of the Swiss "Bundesamt für Veterinärwesen". They were approved by the veterinary office of Canton Basel-Stadt, Switzerland (permission number 739).

### Cluster analysis

The drugs selected in this study were clustered according to certain criteria including a) main indication(s) for which they are registered, b) chemical class and c) mechanisms of action(s). Whenever possible, the DrugBank classification (http://www.drugbank.ca) was followed to assign indication as well as mechanism of action labels to the selected drugs. These labels do not intend to be exhaustive since additional indications as well as mechanisms of action are known for several of the drugs. Chemical classes were arbitrarily defined according the chemical scaffolds of the molecules under consideration, with the exception of protease inhibitors that are better captured under this appellation due to structural variety.

## Results and Discussion

A set of 100 registered drugs were collected (S1 Table) in the framework of DND*i* exploratory activities and submitted systematically to a panel of *in vitro* assays to be profiled for their antiprotozoal activities. These drugs and drug classes were primarily selected for their potential to be repurposed provided that *in vitro* activity could be demonstrated. The inclusion criteria comprised a favorable bioavailability profile, moderate cost of goods and a good safety profile. The selection is heavily biased for anti-infectious indications (66 compounds) including antibiotics (26), antifungals (14), antivirals/antiretrovirals (16) as well as antiparasitic compounds (10) and 15 psychoactive compounds. Another 19 drugs are related to other indications (S1 Table). In some instances drugs were selected based on literature reports of antiprotozoal activity in relation to one specific molecule or class of compounds. A panel of well-known antiprotozoal drugs such as artesunate, mefloquine, pentamidine, nifurtimox and amphotericin B (not an exhaustive list) were included as benchmarks as well as to cross-profile these drugs in the entire screening assay panel.

The results (S2 Table) are ranked in agreement with the *in vitro* activity cutoffs defined at the hit stage for kinetoplastids [14] and for *P*. *falciparum* [15]. Chemical structures and biological data sets of all drugs included as part of this research study are available in the CHEMBL-NTD database https://www.ebi.ac.uk/chemblntd.

### Human African trypanosomiasis

Pentamidine and nifurtimox were, unsurprisingly, identified as active against *T*. *b*. *rhodesiense*; both drugs are used for the treatment of human African trypanosomiasis (HAT) (Table 1, S1 Fig). The mode of action of pentamidine—an aromatic diamidine, a chemical class well-known for its antitrypanosomal activity—is not fully understood. There is evidence that impairment of mitochondrial function is involved [16] and that this family of compounds can rapidly accumulate within trypanosomes as demonstrated with DB75 and DB820 [17]. Nifurtimox is a well-known antitrypanosomal nitrofurane, causing oxidative stress in the target cell [18]. More recently, the activation of nifurtimox by trypanosomal type I nitroreductases leading to the generation of cytotoxic nitrile metabolites has been described [19].

**Table 1:** In vitro activity against T.b. rhodesiense in IC50 (μM) of compounds fulfilling hit criteria.

Drug ID	^a^ *T*. *b*. *rhod*.	^b^Cytotox. L6	^c^SI	Indication	Chemical Class	Mode of Action
Pentamidine	0.01	8.87	887	Antibacterial/Antiprotozoal	Dibenzimides	Interferes with nuclear synthesis/ interfering agent/ DNA, RNA, phospholipids and protein synthesis inhibitor
Auranofin	0.01	4.79	479	Antirheumatic	Gold agent	kappaB kinase and thioredoxin reductase inhibitor
Nifuroxazide	0.03	12.31	410	Antibacterial	Nitroheterocycles	Lipoamide dehydrogenase inhibition
Nitrofurantoin	0.5	90.31	181	Antibacterial	Nitroheterocycles	Oxygen-insensitive NADPH nitroreductase
Thioridazine	0.53	5.39	10	Antipsychotic/Antidepressant	Tricyclics	Dopamine D1 and D2 inhibitor
Amphotericin B	0.76	10.27	14	Antifungal/Antiprotozoal	Polyenes	Membrane cell sterol binder
Sertraline	0.77	8.10	11	Antipsychotic/Antidepressant	Tetrahydro-napthalenamines	Selective serotonin-reuptake inhibitors
Rifamycin SV	0.99	15.68	16	Antibacterial/Antituberculotic	Rifamycins	bacterial DNA-dependent RNA synthesis inhibitor
Paroxetine	1.13	13.84	12	Antipsychotic/Antidepressant	Dehydrophenyl-piperidines	Selective serotonin-reuptake inhibitors
Nortryptyline	1.17	27.87	24	Antipsychotic/Antidepressant	Tricyclics	Serotonin reuptake inhibitor
Triflupromazine	1.42	18.5	13	Antipsychotic/Antiemetic	Tricyclics	Dopamine D1 and D2 receptor inhibitors
Nifurtimox	1.44	87.02	60	Antibacterial/Antiprotozoal	Nitroheterocycles	Induction of oxidative stress in target cell
Clomipramine	2.06	19.79	10	Antipsychotic/Antidepressant	Tricyclics	Serotonin reuptake inhibitor
Promazine	2.16	30.06	14	Antipsychotic/Antidepressant	Tricyclics	Dopamine, serotonine, alpha1 and histamine receptor inhibitor
Amitriptyline	3.03	42.18	14	Antipsychotic/Antidepressant	Tricyclics	Norepinephrine and serotonin reuptake inhibitor
Chloroquine	3.81	50.61	13	Antimalarial	Quinolines	Heme polymerase inhibitor
Pizotifen	3.99	45.02	11	Antimigraine	Tricyclics	serotonin receptor antagonist

^a^ *T*. *b*. *rhod*.:*T*. *b*. *rhodesiense* strain STIB 900, trypomastigotes.

^b^Cytotoxicity on L6 cells.

^c^Selectivity index: IC_50_ Cytotoxicity L6/ IC_50_ *T*. *b*. *rhodesiense*.

IC_50_ values are means of two independent assays, which varied < ±50%.

Two 5-nitrofuran antibiotics chemically related to nifurtimox (S1 Fig), namely nifuroxazide (IC_50_ = 0.03 μM, SI: 410) and nitrofurantoin (IC_50_ = 0.5 μM, SI: 180) were identified as being remarkably potent against *T*. *b*. *rhodesiense* (Table 1). The nitrophenylbenzamine niclosamide showed a lower *in vitro* activity (IC_50_ = 1.67 μM), whereas the 5-nitroimidazole derivatives metronidazole and tinidazole were shown to be inactive in the same assay, presumably because they are not activated *via* enzymatic reduction under the experimental conditions. Overall, the potential for drug repurposing of any nitroheterocycles for HAT heavily depends on their toxicity–and notably genotoxicity/mutagenicity—profile in respect to their efficacy in relevant rodent models, as demonstrated by the successful development of fexinidazole currently in Phase III clinical trials [20, 21].

Rifamycin SV (IC_50_ = 0.99 μM, SI: 16) exhibited a selective activity profile against *T*. *b*. *rhodesiense* (Table 1), whereas other members of the rifamycin family (rifabutin, rifampicin and rifaximin) were devoid of antitrypanosomal activity. Rifamycins have been used for the treatment of several diseases, the most important being HIV-related tuberculosis. Rifamycin SV is a semi-synthetic broad-spectrum antibiotic with activity against Gram-positive and Gram-negative bacteria and mycobacteria. It belongs to the class of ansamycins obtained from rifamycin B, which is produced by fermentation of *Streptomyces mediterranei n*. *sp*. Rifamycin SV is not readily bioavailable and is used parenterally or topically in the treatment of cutaneous and soft tissue infections. Rifamycin SV has rather limited penetration into the brain which is a clear liability for the repositioning of this drug for HAT.

Auranofin showed good and selective activity against *T*. *b*. *rhodesiense* (IC_50_ = 0.01μM, SI: 479) (S1 Fig). Auranofin is a gold complex used to treat rheumatoid arthritis. It putatively acts as an inhibitor of kappa B kinase and thioredoxin reductase which would lead to a decreased immune response and decreased free radical production, respectively [22]. It is a compound that targets selenoproteins in the bloodstream and the procyclic form of *T*. *brucei* [23]. In a recent high-throughput drug screen, high activity against *Entamoeba* was discovered [24]. Auranofin showed 10 times better activity against *Entamoeba histolytica* than the standard drug metronidazole. Given the relatively good bioavailability of auranofin (17–23%) as well as favorable drug exposure in various tissues in rats (terminal half-life of 29 and 43 hours based on blood and serum levels, respectively) following oral administration of a single dose (6.7 mg /kg) [25], we performed an *in vivo* efficacy study in an acutely infected *T*. *brucei* mouse model. However, after daily oral administration of up to 25 mg/kg auranofin over a 4 day period no *in vivo* efficacy was observed regarding reduction of parasitemia or increased survival time (data not presented). This negative outcome could be explained by the lack of a cidal- mechanism of action, or sub-optimal drug exposure *in vivo* in mice due to a different pharmacokinetic profile from that published for rat, or by high protein binding of auranofin.

Two adamantane derivatives were tested one of which, rimantadine, acted selectively (IC_50_ = 13.83 μM SI: 23) against *T*. *b*. *rhodesiense*, albeit at a moderate level. The activity of rimantadine and of other adamantane derivatives against *T*.*brucei* has already been reported by Kelly et al. in 1999 and 2001 [26, 27] and Zoidis et al. 2008 [28]. Ademantanes presumably target essential *T*. *brucei* membrane-localized ion channels or transporters [29, 30]. Adamantanes are inexpensive, orally active drugs [31]. They exhibit steady-state levels in serum of 2.5 to 5.0 μM and plasma half-lives of 24 to 36 hours in humans [32, 33]. Furthermore, adamantanes readily cross the blood-brain barrier [34]. As such adamantanes, and more particularly the *T*. *brucei* active rimantadine, seem to offer promising potential in terms of drug repurposing for HAT, although the moderate *in vitro* potency of rimantadine might be insufficient to demonstrate efficacy *in vivo* given the aforementioned serum levels. Adamantanes may therefore be preferably pursued as part of a lead optimization program to increase potency against *T*. *brucei*. A limited evaluation of 17 adamantanes supported this approach as the most active derivative (1-adamantyl-4-amino-cyclohexane) was about 20 to 25 times more effective than rimantadine [27]. The same study delivered the first proof of principle of efficacy of adamantanes *in vivo*, with a transient 98% suppression of parasitemia in mice with an acute *T*. *brucei* infection. These encouraging results seem to indicate that lead optimization might be more promising than a repurposing strategy for this class of compounds.

A key feature of the TPP in curing the second stage of HAT is CNS penetration [9]. Psychoactive compounds by definition cross the blood-brain barrier. All antidepressant and antipsychotic drugs–including tricyclics and selective serotonin reuptake inhibitors—displayed IC_50_ values in the range of 0.5–2 μM (Table 1) against *T*. *b*. *rhodesiense*, as well as a limited selectivity window with respect to the L-6 rat myoblast cell line apart from nortriptyline (SI> 20) (S2 Fig). These drugs act in various ways and levels on dopaminergic and serotoninergic central receptors indicating that they all have the potential to cross the blood brain barrier. The related drugs thioridazine, triflupromazine, promazine and chlorpromazine are D2 dopamine receptor antagonists and Ca^2+^ channel blockers. Nortriptyline inhibits reuptake of norepinephrine and is a strong antagonist of the H_1_ receptor. It is also known as a Na^+^ channel blocker. There were earlier attempts to develop tricyclic compounds as trypanothione reductase inhibitors *via* lead optimization efforts [35, 36]. However no clear relationship between the activities measured on trypanothione reductase and the *T*. *brucei* whole cell assay could be drawn from a series of 22 inhibitors [36]. It is, to our knowledge, the first time that selective serotonin reuptake inhibitors (including sertraline and paroxetine) are reported to show activity against *T*. *b*. *rhodesiense*. The poly-pharmacology profile of these drugs, notably with respect to associated central effects and toxicity will have to be carefully considered in the light of dose findings in mouse models.

### Chagas’ disease

Not surprisingly nitroheterocycles, in particular nitrofurane derivatives including nifurtimox, nifuroxazide and nitrofurantoin, exhibited the highest antichagasic activity (Table 2, S3 Fig). Nitrofuranes are well known for their antichagasic activity: Nifurtimox—as well as benznidazole, the second treatment available for Chagas’ disease- has been shown to be activated by a NADH-dependent, mitochondrially localized type I nitroreductase [37]. A repurposing strategy for any nitrofurans or nitroimidazole analogues including nifuroxazide and nitrofurantoin, must be based primarily on the safety profile compared to currently used drugs. This notably includes genotoxicity/mutagenicity as previously mentioned in the case of human African trypanosomiasis. In addition, any compound should demonstrate equivalent or better *in vitro* activity and *in vivo* efficacy than the current drugs. Interestingly, another compound from the nitroimidazole class–fexinidazole—has recently also been reported for its oral efficacy in acute and chronic experimental models of benznidazole-susceptible, partially resistant, or resistant *T*. *cruzi* isolates [38] and could therefore be considered as a good candidate for drug repositioning.

**Table 2:** *In vitro* activity against *T*. *cruzi* in IC_50_ (μM) of compounds fulfilling hit criteria.

Drug ID	^a^ *T*. *cruzi*	^b^Cytotox. L6	^c^SI	Indication	Chemical Class	Mode of Action
Bifonazole	0.003	39.30	>1000	Antifungal	Azoles	14alpha-sterol demethylase inhibitor
Itraconazole	0.004	1.11	278	Antifungal	Azoles	14alpha-sterol demethylase inhibitor
Clotrimazole	0.006	2.99	498	Antifungal	Azoles	14alpha-sterol demethylase inhibitor
Miconazole	0.04	15.44	383	Antifungal	Azoles	14alpha-sterol demethylase inhibitor
Econazole	0.04	15.60	390	Antifungal	Azoles	14alpha-sterol demethylase inhibitor
Tioconazole	0.064	19.47	304	Antifungal	Azoles	14alpha-sterol demethylase inhibitor
Ketoconazole	0.27	50.99	189	Antifungal	Azoles	14alpha-sterol demethylase inhibitor
Fluconazole	9.96	>294	>30	Antifungal	Azoles	14alpha-sterol demethylase inhibitor
Nifurtimox	0.19	87.02	458	Antibacterial/Antiprotozoal	Nitroheterocycles	Induction of oxidative stress in target cell
Nifuroxazide	0.23	12.31	54	Antibacterial	Nitroheterocycles	Lipoamide dehydrogenase inhibition
Nitrofurantoine	4.35	90.31	21	Antibacterial	Nitroheterocycles	Oxygen-insensitive NADPH nitroreductase
Mebeverine	3.89	70.77	18	Antispasmotic	Phenylbenzoates	serotonin 5-HT3 receptor antagonist
Tadalafil	8.60	221.1	26	Erectile dysfunction	Pyridoiindolediones	cGMP-specific 3',5'-cyclic phosphodiesterase inhibitor

^a^ *T*. *cruzi*, strain Tulahuen C4, intracellular amastigotes.

^b^Cytotoxicity on L6 cells.

^c^Selectivity index: IC_50_ Cytotoxicity L6/ IC_50_ *T*. *cruzi*.

IC_50_ values are means of two independent assays, which varied < ±50%.

Azoles were identified as the most potent class of inhibitors: six representatives had IC_50_ values in the range of 0.003–0.3 μM and SI: >100 (bifonazole, clotrimazole, econazole nitrate, miconazole and tioconazole as imidazoles as well as itraconazole and ketoconazole as triazoles) while other compounds from this class displayed lower activity and/or selectivity against *T*. *cruzi* (Table 2). These well-known antifungal drugs are already known for their activity against *T*. *cruzi* and for acting *via* inhibition of 14-alpha-sterol demethylase, an enzyme of the sterol biosynthesis pathway [39]. Two triazole antifungals, posaconazole and E1224 (a prodrug of ravuconazole), have recently been reported as failing to demonstrate sustained clearance of *T*. *cruzi* parasitemia in chronically infected patients in phase II clinical trials, putting azoles as a therapeutic class at stake for the treatment of Chagas’disease, at least in monotherapy [40]. This outcome might well be correlated with the inability of azoles and of non-azole CYP51 inhibitors to achieve parasite clearance *in vitro* in various *T*. *cruzi* lineages [41].

Two other compounds that showed moderate micromolar *in vitro* activity against *T*. *cruzi* were tadalafil (IC_50_ = 8.6 μM SI: >26) and mebeverine (IC_50_ = 3.89 μM SI: 18) (Table 2, S3 Fig). Tadalafil is a phosphodiesterase type 5 (PDE5) inhibitor used in treating erectile dysfunction. PDEs are cAMP-specific hydrolases and play a major role in cyclic nucleotide signaling [42]. One of the main challenges to be considered in terms of drug repurposing of PDE inhibitors relates to the safety profile associated with the structural similarity between the human and protozoan PDE. However, the recently identified parasite-specific pocket (P-pocket) in the enzymes of *T*.*cruzi*, *L*. *major* and *T*.*brucei* which is close to the active site might allow the design of parasite-specific inhibitors [43, 44].

The antispasmodic mebeverine is used for the treatment of irritable bowel syndrome (IBS) and associated abdominal cramping. It works by relaxing the muscles in and around the gut. Mebeverine is also a functional inhibitor of acid sphingomyelinase (FIASMA) [45] as well as a serotonin 5-HT3 receptor antagonist. To our knowledge this is the first time that tadalafil and mebeverine are reported to have antichagasic properties. Even if the antitrypanosomal activity is moderate, a more careful evaluation of their activity needs to be conducted to better understand their potential for drug repositioning for Chagas’ disease.

### Leishmaniasis

All selected candidates were tested in two different assays, involving axenic amastigotes and intracellular amastigotes of *L*. *donovani*, respectively. The latter assay used peritoneal mouse macrophages as host cells. Amastigotes in macrophages are currently considered to be more relevant for the visceral disease pathology than axenic amastigotes [46]. For cytotoxicity the compounds were counter-screened against non-infected peritoneal mouse macrophages. Apart from amphotericin B and sitamaquine that can be considered as control drugs in this screening, clofazimine was the only compound exhibiting activity in the *Leishmania donovani* intracellular assay as well as an acceptable level of selectivity (SI ∼10) (Table 3, S4 Fig). Amphotericin B is a polyene antifungal drug displaying either fungistatic or fungicidal activity depending on the drug concentration in body fluids with respect to the susceptibility of the investigated fungal microorganism. The liposomal formulation of amphotericin B (marketed as AmBisome) is currently used as monotherapy for the treatment of visceral leishmaniasis. Amphotericin B binds irreversibly to ergosterol, resulting in disruption of membrane integrity and leakage of intracellular components leading to cell death [47].

**Table 3:** *In vitro* activity against *L*. *donovani* in IC_50_ (μM) of compounds fulfilling hit criteria.

Drug ID	^*a*^ *L*.*don*. axen.	^*b*^ *L*. *don*. intracell	^c^Cytotox. mac.inf.	^d^Cytotox. PMM	^e^SI	Indication	Chemical Class	Mode of Action
Auranofin	0.11	>1.47	4.42	N/A	40	Antirheumatic	Gold agent	kappaB kinase and thioredoxin reductase inhibitor
Amphotericin B	0.34	0.31	32.4	22.39	95	Antifungal/ Antiprotozoal	Polyenes	Membrane cell sterol binder
Ciclopirox olamine	1.64	9.09	20.3	20.27	12	Antifungal	Pyridinones	Polyvalent metal cation chelator
Tolnaftate	4.33	50.1	97.6	N/A	>23	Antifungal	Thiocarbamates	Squalene epoxidase inhibitor
Artesunate	0.35	>7.8	7.8	N/A	>22	Antimalarial	Endoperoxides	Unknown, acting *via* reactive oxygen radical species
Rifamycin SV	1.5	>13.87	41.62	N/A	28	Antibacterial/ Antituberculotic	Rifamycins	bacterial DNA-dependent RNA synthesis inhibitor
Rifampicin	1.53	>36.45	36.5	N/A	>24	Antibacterial/ Antituberculotic	Rifamycins	Bacterial DNA-dependent RNA synthesis inhibitor
Nitrofurantoine	2.12	>41.81	125.44	N/A	59	Antibacterial	Nitroheterocycles	Oxygen-insensitive NADPH nitroreductase
Nifurtimox	2.76	20.68	34.8	15.7	13	Antibacterial/ Antiprotozoal	Nitroheterocycles	Induction of oxidative stress in target cells
Troglitazone	4.26	>67.94	68	N/A	>16	Antidiabetic/ Antinflammatory	Thiazolidinediones	Nuclear receptor (PPAR) binder
Clofazimine	22.39	0.95	6.34	10.65	10	Antibacterial/ Antituberculotic	Riminophenazines	Mycobacterial DNA binder, Redox cycling, Cell membrane destabilizer, Acid sphingomyelinase inhibitor
Nifuroxazide	2.83	>10.86	36.2	N/A	13	Antibacterial	Nitroheterocycles	Lipoamide dehydrogenase inhibition
Tipranavir	1.64	>49.78	50	N/A	>30	Antiviral/ Antiretroviral	Protease Inhibitors	HIV protease inhibitor
Lonidamine	8.66	>93.41	93.4	N/A	>11	Anticancer	Indazoles	Glycolysis inhibition *via* hexokinase activation

^*a*^ *L*. *don*. axen.: axenic amastigotes of *L*. *donovani*, strain MHOM-ET-67/L82.

^*b*^ *L*. *don*. intracell: intracellular amastigotes of *L*. *donovani* strain MHOM-ET-67/L82.

^c^Cytotoxicity on macrophages infected with *L*. *donovani*.

^d^Cytotoxicity on peritoneal mouse macrophages.

^e^Selectivity index: IC_50_ Cytotoxicity macrophages/ IC_50_ *L*. *donovani*. IC_50_ values are means of two independent assays, which varied < ±50%.

Sitamaquine, a known antileishmanial drug, displayed only moderate activity against both axenic and intracellular amastigotes (Table 3). The drug development of sitamaquine was discontinued in Phase II clinical trials by GlaxoSmithKline due to safety concerns related to methemoglobinemia, a known feature of 8-aminoquinolines [48].

Clofazimine is a lipophilic riminophenazine derivative possessing both antimycobacterial and anti-inflammatory properties. Its efficacy has been demonstrated in the treatment of leprosy only in combination with rifampicin and dapsone, but not in human tuberculosis, despite the fact that it is impressively active *in vitro* against multidrug-resistant strains of *Mycobacterium tuberculosis* [49]. Interestingly, clofazimine is more active against intracellular than axenic *Leishmania donovani*, putatively due to the accumulation of clofazimine in the macrophages, a known feature of riminophenazines [50]. The antileishmanial properties of clofazimine have previously been reported both *in vitro* and in animal models for three different *Leishmania* species including *L*. *donovani* [51]. Clofazimine binds to guanine bases leading to an inhibition of cell proliferation [52]. Additionally, clofazimine inhibits acid sphingomyelinase (FIASMA) and increases the activity of phospholipase A2 [47]. Cell membrane destabilization and subsequent dysfunction as well as intracellular redox cycling involving oxidation of reduced clofazimine leading to the generation of reactive oxygen species were proposed as mechanisms contributing to the antimycobacterial activity of clofazimine. These putative mechanisms of action have recently been reviewed by Cholo et al. 2012 [49]. Considering the very good pharmacokinetic, distribution and safety profiles of clofazimine in the mouse [50] it seems quite reasonable to envisage an *in vivo* efficacy study of this drug in a relevant mouse model infected with *Leishmania donovani*.

Tipranavir (a non-peptidic protease inhibitor [53]), the antimalarial artesunate and other antibacterials like nitrofurantoine, nifuroxazide, rifampicin and rifamycin SV were all active (IC_50_: < 3μM) against axenic amastigotes of *L*. *donovani*, but inactive against the intracellular amastigotes or cytotoxic on the host cell (Table 3). Auranofin was active against axenic amastigotes (IC_50_ = 0.11μM). But auranofin did not exhibit activity against intracellular amastigotes at a concentration of 1.47 μM and at 4.42 μM it was cytotoxic on the host cells. This is somewhat contradictory to published data [54], which however used different Leishmaia species and a different host cell. The hydroxypyridinone antifungal ciclopirox olamine showed activity against axenic amastigotes and activity against intracellular amastigotes of *L*. *donovani* (IC_50_ = 0.1 μM, SI: 9) with moderate selectivity (Table 3, S4 Fig). In addition, the two azoles clotrimazole and tioconazole were active with low selectivity against intracellular *L*. *donovani* (S1 Table). Niclosamide used as an anthelmintic, in addition to auranofin, showed the best activity of all tested compounds against *L*. *donovani* axenic amastigotes but it was inactive against intracellular amastigotes at a concentration of 0.1 μg/ml, and toxic to mouse macrophages at higher concentrations (>0.3 μg/ml) (S1 Table). The repurposing potential of these few drugs seems rather low as they were either not able to demonstrate any significant activity in the intracellular *L*. *donovani* assay or alternatively lacked selectivity.

### Malaria

The *in vitro* activity of all of the tested standard animalarials (artesunate, mefloquine, tafenoquine, chloroquine and sitamaquine) was confirmed against *P*. *falciparum* as shown in Table 4. Interestingly, four of the tested azoles (clotrimazole, econazole, miconazole and tioconazole) were active against *P*. *falciparum* (Table 4, S5 Fig) confirming the finding of Penna Coutinho et al. 2011 [55] who described the antimalarial activity of posaconazole and itraconazole.

**Table 4:** *In vitro* activity against *P*. *falciparum* in IC_50_ (μM) of compounds fulfilling hit criteria.

Drug ID	^*a*^ *P*. *falc*. K1	^b^Cytotox. L6	^c^SI	Indication	Chemical Class	Mode of Action
Mefloquine	0.002	3.25	1354	Antimalarial	Quinolines	Unknown, putative heme polymerase inhibitor
Artesunate	0.003	0.78	260	Antimalarial	Endoperoxides	Unknown, acting *via* reactive oxygen radicals
Chloroquine	0.17	50.61	298	Antimalarial	Quinolines	Heme polymerase inhibitor
Tafenoquine	0.27	5.52	20	Antimalarial	Quinolines	Unknown, putative heme polymerase inhibitor
Sitamaquine	0.08	32.31	404	Antileishmanial	Quinolines	Unknown
Rifampicin	0.1	75.22	752	Antibacterial/Antituberculotic	Rifamycins	Bacterial DNA-dependent RNA synthesis inhibitor
Rifamycin SV	0.55	15.68	29	Antibacterial/Antituberculotic	Rifamycins	bacterial DNA-dependent RNA synthesis inhibitor
Rifaximin	0.92	88.05	96	Antibacterial/Antituberculotic	Rifamycins	Bacterial DNA-dependent RNA synthesis inhibitor
Amphotericin B	0.8	10.27	13	Antifungal/Antiprotozoal	Polyenes	Membrane cell sterol binder
Clotrimazole	0.11	2.99	27	Antifungal	Azoles	14alpha-sterol demethylase inhibitor
Econazole	0.32	15.6	49	Antifungal	Azoles	14alpha-sterol demethylase inhibitor
Miconazole	0.49	15.44	32	Antifungal	Azoles	14alpha-sterol demethylase inhibitor
Tioconazole	0.63	19.47	31	Antifungal	Azoles	14alpha-sterol demethylase inhibitor
Promazine	0.49	30.06	61	Antipsychotic/Antidepressant	Tricyclics	Dopamine, serotonin, alpha1 and histamine receptor inhibitor
Fluphenazine	0.50	11.54	23	Antipsychotic/Antidepressant	Tricyclics	Dopamine receptor inhibitor
Sertraline	0.51	8.10	16	Antipsychotic/Antidepressant	Tetrahydro-napthalenamines	Selective serotonin-reuptake inhibitors
Nortryptyline	0.58	27.87	48	Antipsychotic/Antidepressant	Tricyclics	Serotonin reuptake inhibitor
Ketotifen	0.75	147.04	196	Antihistamine	Cycloheptathio-phenones	H1-Histamine receptor antagonist
Cloperastine	0.87	43.35	50	Cough Suppressant	Phenylmethoxy-piperidines	Unknown
Rimantadine	0.97	311.2	321	Antiviral/Antiretroviral	Adamantanes	Matrix protein 2 inhibitor

^*a*^ *P*. *falc*.: *P*. *falciparum* strain K1.

^b^Cytotoxicity on L6 cells.

^c^Selectivity index: IC_50_ Cytotoxicity L6/ IC_50_ *P*. *falciparum*.

IC_50_ values are means of two independent assays, which varied < ±50%.

Rifamycins, especially rifampicin (IC_50_ = 0.1 μM, SI: >100), showed remarkably selective activity in the antiplasmodial assay (Table 4). The anti-tuberculosis drug rifampicin is an RNA polymerase inhibitor of bacterial transcription and was previously described for its *in vitro* and *in vivo* antimalarial activities [56, 57]. To our knowledge, other compounds from this class have not been reported to have antimalarial activity.

The antiplasmodial activity associated with tricyclic antidepressants (Table 4, S5 Fig) is certainly one of the most striking observations of this screen. Promazine and nortriptyline displayed the highest selective activity against *P*. *falciparum*. Promazine is a phenothiazine compound and a D2 dopamine receptor antagonist which showed an IC_50_ value of 0.49 μM with a selectivity index of 61. Nortriptyline, a tricyclic antidepressant and potent inhibitor of the norepinephrine transporter exhibited an IC_50_ value of 0.58 μM against *P*. *falciparum*, and a selectivity index of 48. Tricyclic antidepressant drugs have previously been shown to reverse chloroquine resistance in *P*. *falciparum in vitro* and in monkey studies [58].and were additionally described in a recent publication as blocking agents for Plasmodium oocyst development and transmission [59]. Transmission blocking is an important feature for the elimination of malaria. It is worth noting that further tricyclics (including fluphenazine and amitriptyline) as well as selective serotonin reuptake inhibitors (sertraline and fluoxetine) also displayed antiplasmodial activities in the micromolar range. Additionally, *in vitro* selective activities against *P*. *falciparum* were identified for the antiviral rimantadine, the anti-thrombotic dipyridamole, the anti-tussive clopersatine, and the anti-histamine ketotifen. All of these activities have already been reported elsewhere [60, 61]. Providing a cidal mechanism of action can be confirmed for these drugs, the next step will consist of an evaluation of their potential to suppress parasitemia in a mouse malaria model. If successful, the repurposing potential of these drugs will need to be carefully assessed considering the safety profile at the defined curative dose, notably in relation to the pharmacological effects of these drugs at the dosing regimen used. This constitutes a major challenge, especially for the drugs for which there is a dramatic discrepancy in terms of *in vitro* activities between their primary indication (generally 1–10 nM range) and malaria (100 nM-1μM range). The compatibility of these drugs with a short (1–3 day) oral treatment, their low susceptibility to generate resistance, and their amenability for use in combination with existing antimalarial drugs will similarly need to be considered.

## Conclusion

Several drugs and drug classes were confirmed to have *in vitro* activity against the four protozoan parasites *T*. *brucei rhodesiense*, *L*. *donovani*, *T*. *cruzi* and *P*. *falciparum*, offering various opportunities for drug repurposing. Several of these antiparasitic activities–but not all- have already been reported. To our knowledge it is indeed the first time that tadalafil and mebeverine have been described for their antichagasic activity. For these drugs a wealth of preclinical and clinical data can be used to determine whether their safety profiles are compatible with the anticipated dose of drug to be used in animal models and eventually in patients. The candidates for further development should ideally be associated with a favorable bioavailability profile, as oral drug administration is preferable for the next generation of drugs used to treat kinetoplastid diseases. The reasons for a lack of or insufficient *in vi*vo efficacy in relevant preclinical animal models shall be further investigated to assist in the decision to drop or further pursue an existing drug for repurposing. There is a clear need to carefully define the types of preclinical experiments that need to be run to progress the candidates identified from screening in the framework of a defined drug discovery cascade supported by DMPK and toxicity assays.

Drug repurposing is a discovery strategy that aims to maximise pre-existing preclinical and clinical knowledge accumulated on registered drugs and drug candidates for a new indication [12], and is nowadays actively pursued by pharmaceutical companies [62] and currently accounts for approximately 30% of the newly approved drugs and vaccines by the US Food and Drug Administration—in recent years [63]. The area of neglected diseases has counted for a few drug repositioning successes such as the antibacterial sulfonamides (dapsone, sulfadoxine), tetracyclines (doxycycline) and combination of trimethoprim/sulfamethoxazole for malaria [64], fluoroquinolones for tuberculosis [64], and the anticancer agent miltefosine as well as the antifungal amphotericin B for the treatment of visceral leishmaniasis [65].

Several approaches can be used to address the identification of novel drug candidates at an early discovery stage using a drug repositioning approach. They notably include target-based screening, phenotypic (“target unbiased or blinded”) screening, knowledge-based methods (e.g. chemoinformatics and bioinformatics), signature-based methods, pathway or network methods and targeted mechanism-based methods, reviewed and illustrated elsewhere [63]. From a recent comparative analysis based on 259 approved agents [66], 50 were shown to be first-in-class small molecules associated with a new molecular mechanism of action, of which 28 and 17 of these drugs were identified from phenotypic screening and target-based approaches, respectively. These results illustrate the impressive potential of phenotypic screening in the area of drug discovery. The screening of a library of drugs and drug candidates in a phenotypic assay is therefore seen as an attractive way to identify new potential candidates with a modest work load. This can be illustrated by the discovery of the antimalarial properties of astemizole from the screening of 2687 approved drugs or drug candidates using a *P*. *falciparum* whole cell *in vitro* screening assay [67].

In summary, this low-hanging fruit approach is certainly worth the effort in a “low risk, high return on investment” drug discovery process, especially in the field of neglected tropical diseases where R&D has to be addressed with limited resources. The availability of a significant amount of data and expertise can indeed lead to significant savings in terms of time and money. Some of the approved marketed drugs will have the additional advantage of being off-patent, facilitating the drug repurposing process from an intellectual property management standpoint. A drawback related to the progression of old drugs might however be the lack or the paucity of recently generated data reports (e.g. lack of quality of pharmacokinetic measurements based on LC/MS, and toxicity assays performed in obsolete *in vitro* and *in vivo* predictive models).

## Supporting Information

S1 Fig HAT cluster of all tested compounds. Chemical class vs log (IC_50_ in µM).
(TIFF)

S2 Fig HAT cluster of antidepressant and antipsychotics. Chemical class vs log(IC_50_ in µM).
(TIFF)

S3 Fig Chagas disease cluster of all tested compounds. Chemical class vs log(IC_50_ in µM).
(TIFF)

S4 Fig Leishmaniasis cluster of all tested compounds. Chemical class vs log(IC_50_ in µM).
(TIFF)

S5 Fig Malaria cluster of all tested compounds. Chemical class vs log(IC_50_ in µM).
(TIFF)

S1 Table Table 1. Set of 100 registered drugs tested for their antiparasitic activity.
(DOCX)

S2 Table Table 2. *In vitro* activity profile in IC_50_ (µM) of all tested compounds.
(DOCX)
