# Supplementary figures and images for: Antiprotozoal Activity Profiling of Approved Drugs: A Starting Point toward Drug Repositioning
Source: PLoS One. 2015 Aug 13;10(8):e0135556. doi: 10.1371/journal.pone.0135556 (PMC4535766; doi:10.1371/journal.pone.0135556)

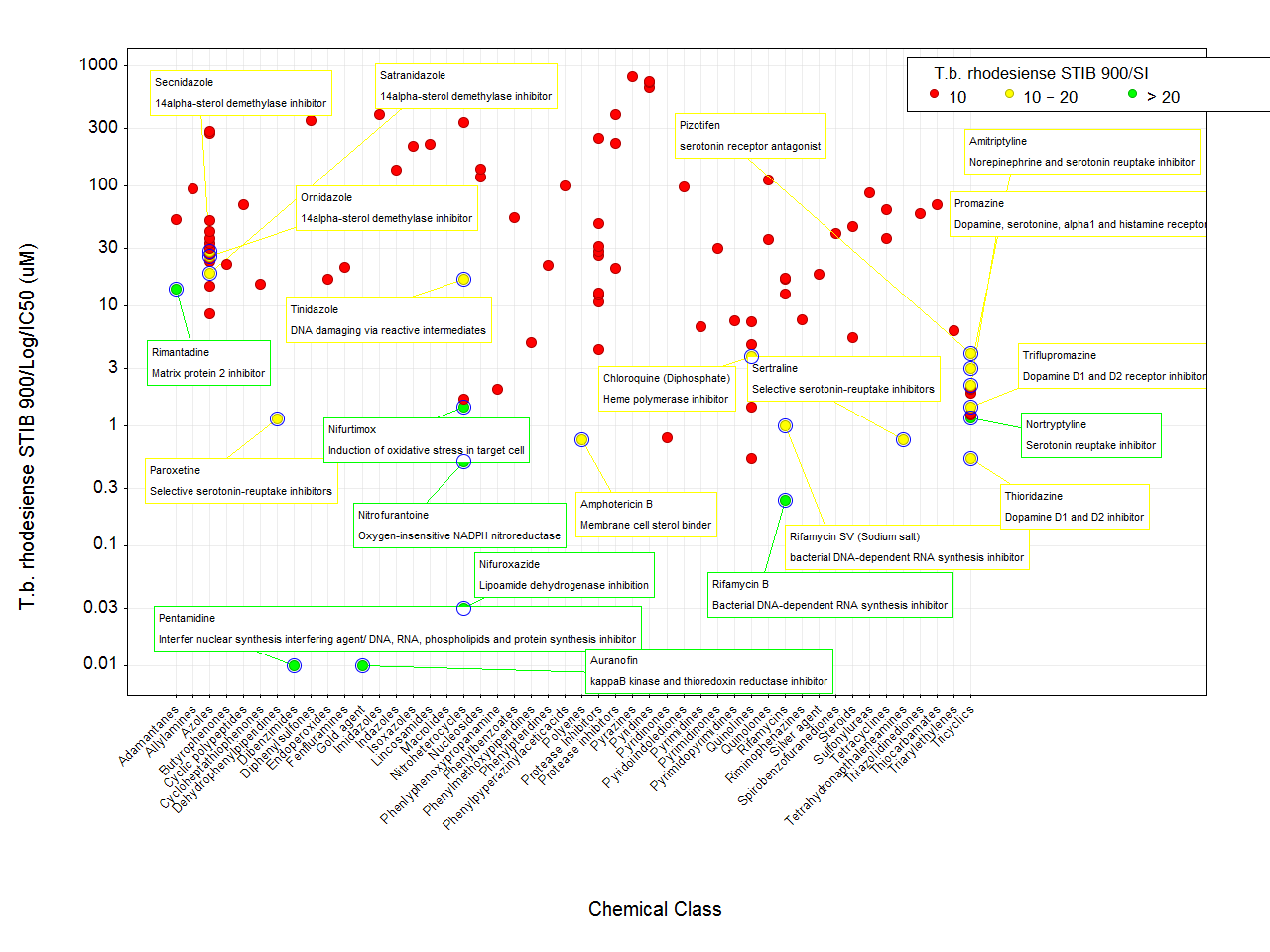

Supplement: S1 Fig — Chemical class vs log (IC50 in µM). (TIFF) [file pone.0135556.s001.tiff]

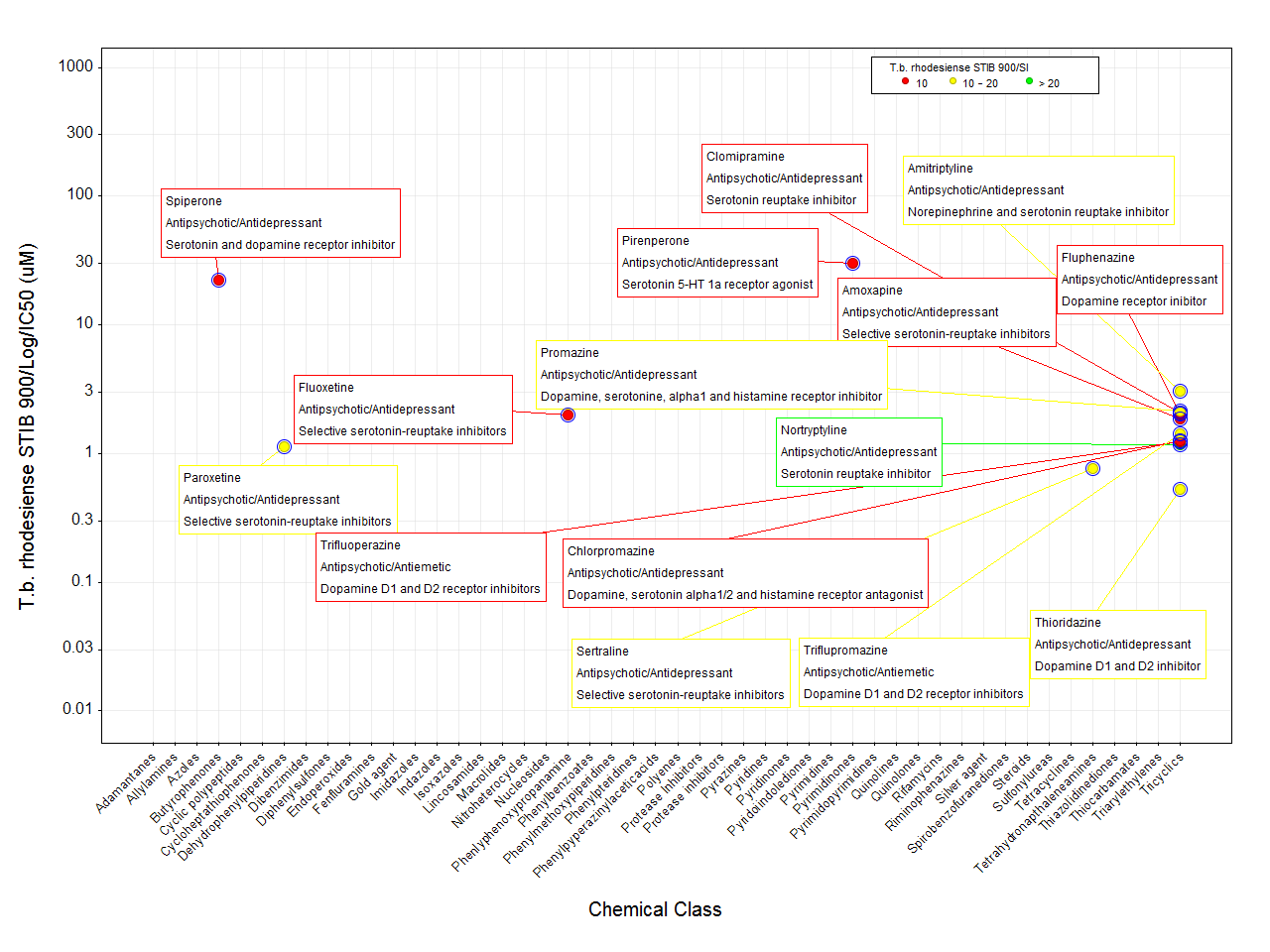

Supplement: S2 Fig — Chemical class vs log(IC50 in µM). (TIFF) [file pone.0135556.s002.tiff]

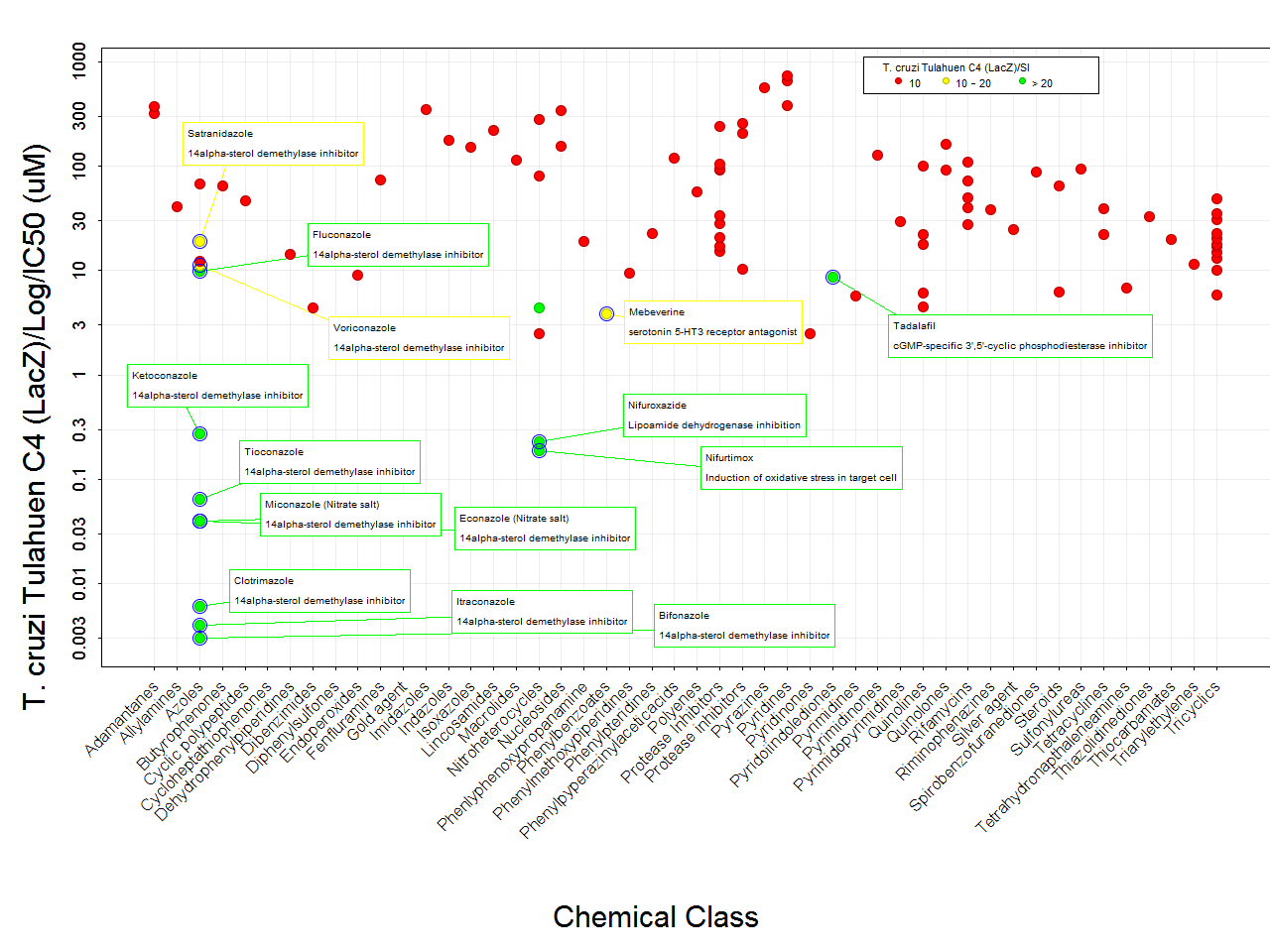

Supplement: S3 Fig — Chemical class vs log(IC50 in µM). (TIFF) [file pone.0135556.s003.tiff]

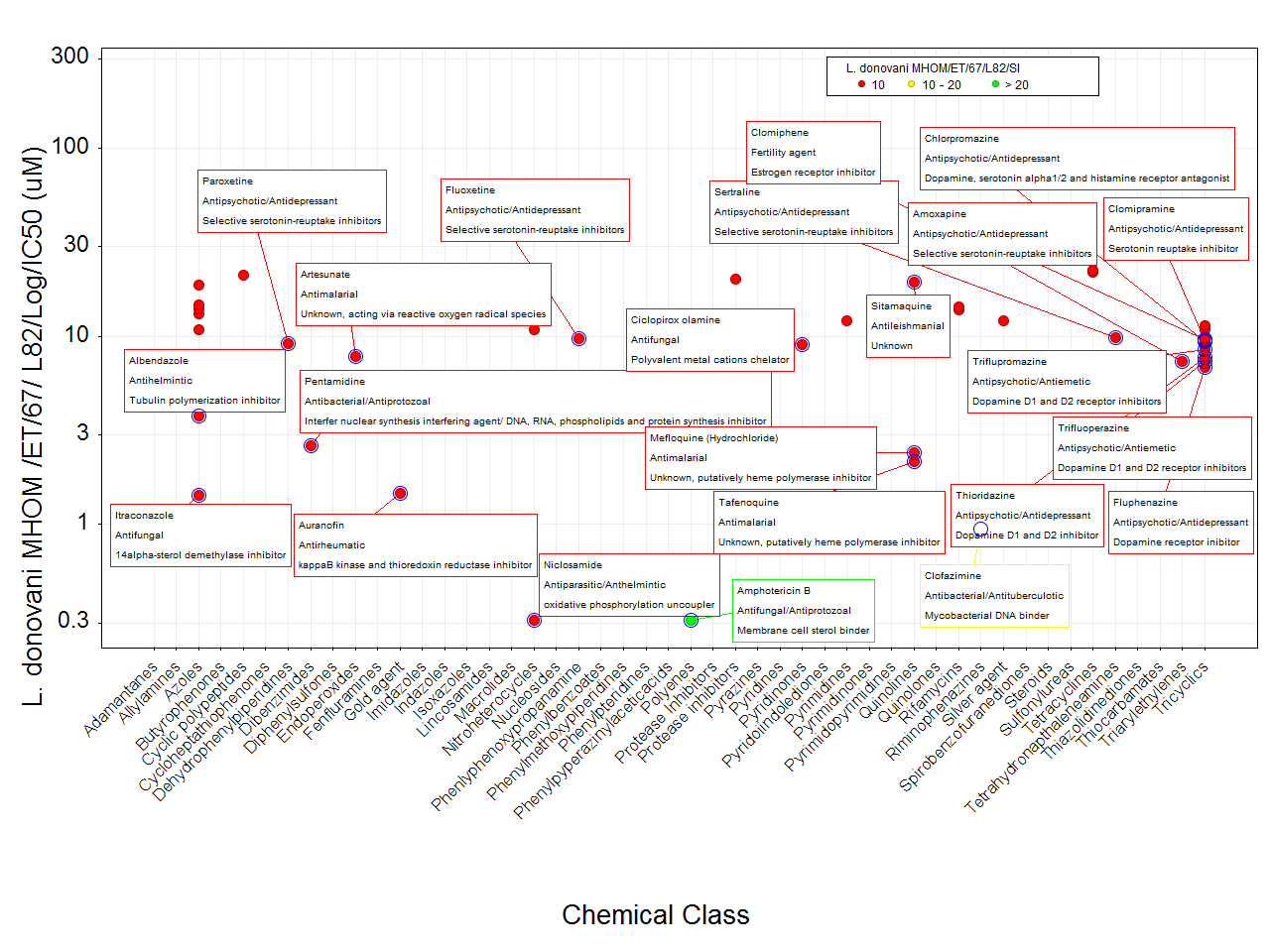

Supplement: S4 Fig — Chemical class vs log(IC50 in µM). (TIFF) [file pone.0135556.s004.tiff]

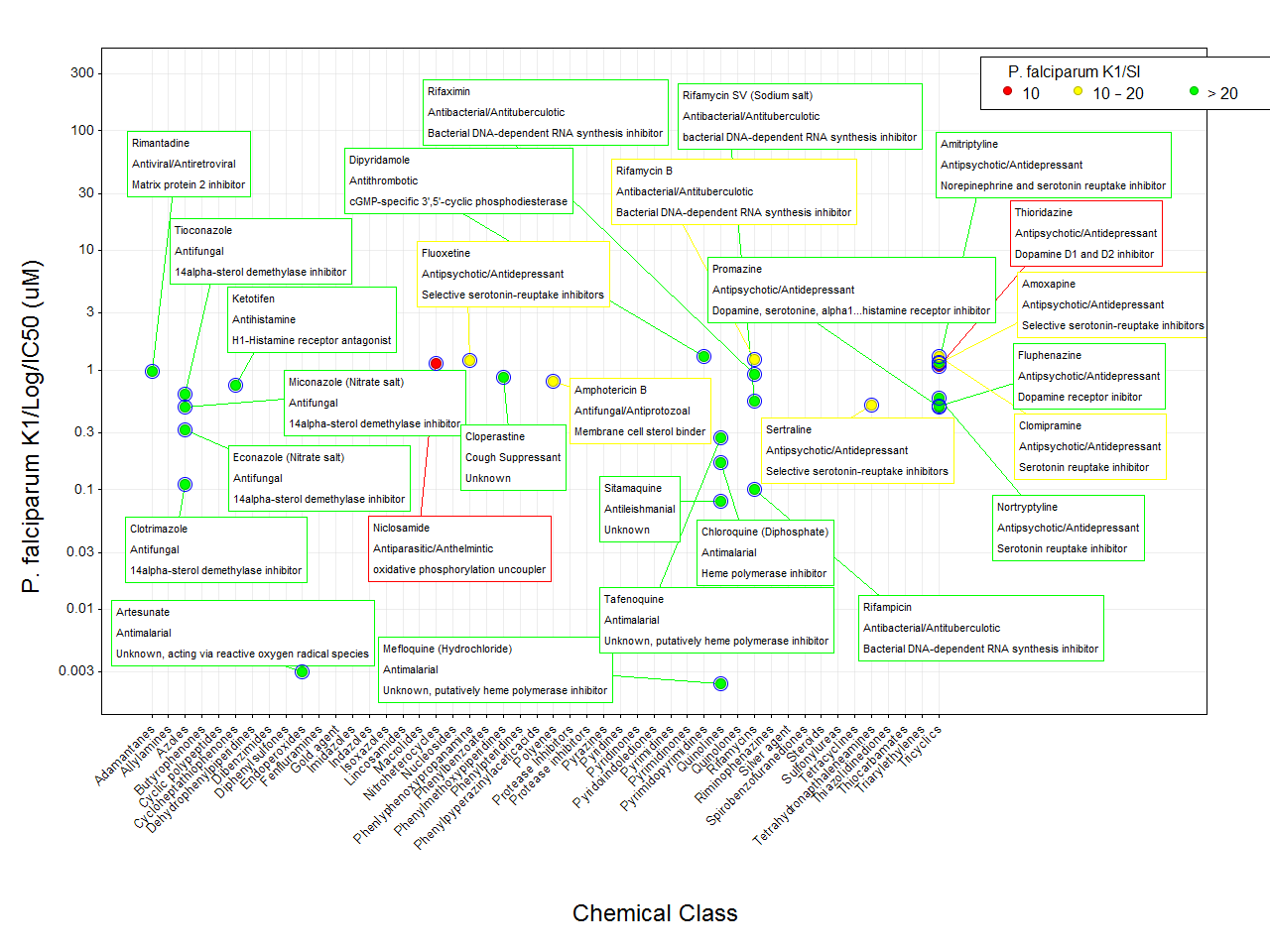

Supplement: S5 Fig — Chemical class vs log(IC50 in µM). (TIFF) [file pone.0135556.s005.tiff]
