# Supplementary material for: Antiprotozoal Activity Profiling of Approved Drugs: A Starting Point toward Drug Repositioning
Source: PLoS One. 2015 Aug 13;10(8):e0135556. doi: 10.1371/journal.pone.0135556 (PMC4535766; doi:10.1371/journal.pone.0135556)
Supplement: S1 Table — Set of 100 registered drugs tested for their antiparasitic activity. (DOCX) [file pone.0135556.s006.docx]

S1 Set of 100 registered drug tested for their antiparasitic activity

| **Drug ID** | **Indication** | **Chemical Class** | **Mode of Action** |
| --- | --- | --- | --- |
| Rimantadine | Antiviral/Antiretroviral | Adamantanes | Matrix protein 2 inhibitor |
| Amantadine | Antiviral/Antiretroviral | Adamantanes | Matrix protein 2 inhibitor |
| Terbinafine (Hydrochloride) | Antifungal | Allylamines | Squalene epoxidase inhibitor |
| Tioconazole | Antifungal | Azoles | 14alpha-sterol demethylase inhibitor |
| Ketoconazole | Antifungal | Azoles | 14alpha-sterol demethylase inhibitor |
| Bifonazole | Antifungal | Azoles | 14alpha-sterol demethylase inhibitor |
| Satranidazole | Antibacterial/Antiprotozoal | Azoles | 14alpha-sterol demethylase inhibitor |
| Secnidazole | Antibacterial/Antiprotozoal | Azoles | 14alpha-sterol demethylase inhibitor |
| Ornidazole | Antibacterial/Antiprotozoal | Azoles | 14alpha-sterol demethylase inhibitor |
| Itraconazole | Antifungal | Azoles | 14alpha-sterol demethylase inhibitor |
| Clotrimazole | Antifungal | Azoles | 14alpha-sterol demethylase inhibitor |
| Albendazole | Antihelmintic | Azoles | Tubulin polymerization inhibitor |
| Econazole (Nitrate salt) | Antifungal | Azoles | 14alpha-sterol demethylase inhibitor |
| Voriconazole | Antifungal | Azoles | 14alpha-sterol demethylase inhibitor |
| Miconazole (Nitrate salt) | Antifungal | Azoles | 14alpha-sterol demethylase inhibitor |
| Fluconazole | Antifungal | Azoles | 14alpha-sterol demethylase inhibitor |
| Omeprazole | Antiulcer agent | Azoles | Proton pump inihibitor |
| Spiperone | Antipsychotic/Antidepressant | Butyrophenones | Serotonin and dopamine receptor inhibitor |
| Bacitracine | Antibacterial | Cyclic polypeptides | Insulin-degrading enzym |
| Ketotifen | Antihistamine | Cycloheptathiophenones | H1-Histamine receptor antagonist |
| Paroxetine | Antipsychotic/Antidepressant | Dehydrophenylpiperidines | Selective serotonin-reuptake inhibitors |
| Pentamidine | Antibacterial/Antiprotozoal | Diamidines | DNA, RNA, phospholipids and protein synthesis inhibitor |

S1 continued

| **Drug ID** | **Indication** | **Chemical Class** | **Mode of Action** |
| --- | --- | --- | --- |
| Dapsone | Antibacterial/Antileprotic | Diphenylsulfones | Dihydrofolic acid synthesis inhibitor |
| Artesunate | Antimalarial | Endoperoxides | Unknown, acting via reactive oxygen radical species |
| Benfluorex | Anorectic/Hypolipidemic | Fenfluramines | Lipase stimulator; PPAR agonist |
| Auranofin | Antirheumatic | Gold agent | kappaB kinase and thioredoxin reductase inhibitor |
| Cimetidine | Antihistamine | Imidazoles | Histamine H2-receptor antagonist |
| Lonidamine | Anticancer | Indazoles | Glycolysis inhibition via hexokinase activation |
| Leflunomide | Antirheumatic | Isoxazoles | Unknown |
| Lincomycin | Antibacterial | Lincosamides | Ribosomal protein synthesis inhibitor |
| Erythromycine (Hydrate) | Antibacterial | Macrolides | Ribosomal protein synthesis inhibitor |
| Nitrofurantoine | Antibacterial | Nitroheterocycles | Oxygen-insensitive NADPH nitroreductase |
| Nifuroxazide | Antibacterial | Nitroheterocycles | Lipoamide dehydrogenase inhibition |
| Metronidazole | Antibacterial/Antiparasitic | Nitroheterocycles | Oxygen-insensitive NADPH nitroreductase |
| Nifurtimox | Antibacterial/Antiprotozoal | Nitroheterocycles | Induction of oxidative stress in target cell |
| Tinidazole | Antibacterial/Antiprotozoal | Nitroheterocycles | DNA damaging via reactive intermediates |
| Niclosamide | Antiparasitic/Anthelmintic | Nitroheterocycles | oxidative phosphorylation uncoupler |
| Zidovudine | Antiviral/Antiretroviral | Nucleosides | Nucleoside reverse transcriptase inhibitor |
| Stavudine | Antiviral/Antiretroviral | Nucleosides | Nucleoside reverse transcriptase inhibitor |
| Fluoxetine | Antipsychotic/Antidepressant | Phenlyphenoxypropanamine | Selective serotonin-reuptake inhibitors |
| Mebeverine | Antispasmotic | Phenylbenzoates | serotonin 5-HT3 receptor antagonist |
| Cloperastine | Cough Suppressant | Phenylmethoxypiperidines | Unknown |
| Triamterene | Diuretic | Phenylpteridines | Epithelial sodium channel inhibitor |
| Cetirizine (Hydrochloride) | Antihistamine | Phenylpyperazinylaceticacids | Histamine H1-receptor inhibitor |
| Amphotericin B | Antifungal/Antiprotozoal | Polyenes | Membrane cell sterol binder |
| Indinavir (Sulfate) | Antiviral/Antiretroviral | Protease Inhibitors | HIV protease inhibitor |

S1 continued

| **Drug ID** | **Indication** | **Chemical Class** | **Mode of Action** |
| --- | --- | --- | --- |
| Ritonavir | Antiviral/Antiretroviral | Protease Inhibitors | HIV protease inhibitor |
| Amprenavir | Antiviral/Antiretroviral | Protease Inhibitors | HIV protease inhibitor |
| Tipranavir | Antiviral/Antiretroviral | Protease Inhibitors | HIV protease inhibitor |
| Ganciclovir | Antiviral/Antiretroviral | Protease Inhibitors | Thymidine kinase activator, DNA polymerase inhibitor |
| Atazanavir (Sulfate) | Antiviral/Antiretroviral | Protease Inhibitors | HIV protease inhibitor |
| Saquinavir | Antiviral/Antiretroviral | Protease Inhibitors | HIV protease inhibitor |
| Darunavir | Antiviral/Antiretroviral | Protease Inhibitors | HIV protease inhibitor |
| Lopinavir | Antiviral/Antiretroviral | Protease Inhibitors | HIV protease inhibitor |
| Nelfinavir | Antiviral/Antiretroviral | Protease inhibitors | HIV protease inhibitor |
| Famciclovir | Antiviral/Antiretroviral | Protease inhibitors | Thymidine kinase activator, DNA polymerase inhibitor |
| Penciclovir | Antiviral/Antiretroviral | Protease inhibitors | Thymidine kinase activator, DNA polymerase inhibitor |
| Pyrazinamide | Antibacterial/Antituberculotic | Pyrazines | Fatty acid synthetase I inhibitor |
| Izoniazide | Antibacterial/Antituberculotic | Pyridines | Micolic acid synthesis inhibition |
| Isoniazide | Antibacterial/Antituberculotic | Pyridines | Miclic acid synthesis inhibition |
| Nicotinamide | Vitamin | Pyridines | (N/A) |
| Ciclopirox olamine | Antifungal | Pyridinones | Polyvalent metal cations chelator |
| Tadalafil | Erectile dysfunction | Pyridoindolediones | cGMP-specific 3',5'-cyclic phosphodiesterase inhibitor |
| Pyrimethamine | Antimalarial | Pyrimidines | Dihydrofolate reductase inhibitor |
| Pirenperone | Antipsychotic/Antidepressant | Pyrimidinones | Serotonin 5-HT 1a receptor agonist |
| Dipyridamole | Antithrombotic | Pyrimidopyrimidines | cGMP-specific 3',5'-cyclic phosphodiesterase |

S1 continued

| **Drug ID** | **Indication** | **Chemical Class** | **Mode of Action** |
| --- | --- | --- | --- |
| Tafenoquine | Antimalarial | Quinolines | Unknown, putatively heme polymerase inhibitor |
| Mefloquine (Hydrochloride) | Antimalarial | Quinolines | Unknown, putatively heme polymerase inhibitor |
| Primaquine | Antimalarial/Antiprotozoal | Quinolines | Unknown, putatively reactive oxygen species or electron transport interference |
| Sitamaquine | Antileishmanial | Quinolines | Unknown |
| Chloroquine (Diphosphate) | Antimalarial | Quinolines | Heme polymerase inhibitor |
| Ciprofloxacin | Antibacterial | Quinolones | DNA gyrase subunit A inhibitor |
| Enoxacin | Antibacterial | Quinolones | DNA gyrase subunit A inhibitor |
| Rifabutin | Antibacterial/Antituberculotic | Rifamycins | Bacterial DNA-dependent RNA synthesis inhibitor |
| Rifampicin | Antibacterial/Antituberculotic | Rifamycins | Bacterial DNA-dependent RNA synthesis inhibitor |
| Rifaximin | Antibacterial/Antituberculotic | Rifamycins | Bacterial DNA-dependent RNA synthesis inhibitor |
| Rifamycin SV (Sodium salt) | Antibacterial/Antituberculotic | Rifamycins | bacterial DNA-dependent RNA synthesis inhibitor |
| Clofazimine | Antibacterial/Antituberculotic | Riminophenazines | Mycobacterial DNA binder, Redox cycling, Cell membrane destabilizer, Acid sphingomyelinase inhibitor |
| Silver sulfadiazine | Antibacterial | Silver agent | Cell membrane interfering agent |
| Griseofulvin | Antifungal | Spirobenzofuranediones | Tubulin binder |
| Danazol | Endomitriosis | Steroids | Gonadotropin inhibitor |
| Ganaxolone | Anesthetic | Steroids | GABAA receptor modulator |
| Glybenclamide | Antidiabetic | Sulfonylureas | [Sulfonylurea receptor 1 activator](http://en.wikipedia.org/wiki/Sulfonylurea_receptor) |
| Doxycycline | Antibacterial | Tetracyclines | Ribosomal protein synthesis inhibitor |

S1 continued

| **Drug ID** | **Indication** | **Chemical Class** | **Mode of Action** |
| --- | --- | --- | --- |
| Minocycline | Antibacterial | Tetracyclines | Ribosomal protein synthesis inhibitor |
| Sertraline | Antipsychotic/Antidepressant | Tetrahydronapthaleneamines | Selective serotonin-reuptake inhibitors |
| Troglitazone | Antidiabetic/ Antinflammatory | Thiazolidinediones | Nuclear receptors (PPAR) binder |
| Tolnaftate | Antifungal | Thiocarbamates | Squalene epoxidase inhibitor |
| Clomiphene | Fertility agent | Triarylethylenes | Estrogen receptor inhibitor |
| Thioridazine | Antipsychotic/Antidepressant | Tricyclics | Dopamine D1 and D2 inhibitor |
| Triflupromazine | Antipsychotic/Antiemetic | Tricyclics | Dopamine D1 and D2 receptor inhibitors |
| Amoxapine | Antipsychotic/Antidepressant | Tricyclics | Selective serotonin-reuptake inhibitors |
| Fluphenazine | Antipsychotic/Antidepressant | Tricyclics | Dopamine receptor inhibitor |
| Clomipramine | Antipsychotic/Antidepressant | Tricyclics | Serotonin reuptake inhibitor |
| Nortryptyline | Antipsychotic/Antidepressant | Tricyclics | Serotonin reuptake inhibitor |
| Promazine | Antipsychotic/Antidepressant | Tricyclics | Dopamine, serotonine, alpha1 and histamine receptor inhibitor |
| Chlorpromazine | Antipsychotic/Antidepressant | Tricyclics | Dopamine, serotonin alpha1/2 and histamine receptor antagonist |
| Amitriptyline | Antipsychotic/Antidepressant | Tricyclics | Norepinephrine and serotonin reuptake inhibitor |
| Trifluoperazine | Antipsychotic/Antiemetic | Tricyclics | Dopamine D1 and D2 receptor inhibitors |
| Pizotifen | Antimigraine | Tricyclics | serotonin receptor antagonist |
