# Supplementary material for: Antiprotozoal Activity Profiling of Approved Drugs: A Starting Point toward Drug Repositioning
Source: PLoS One. 2015 Aug 13;10(8):e0135556. doi: 10.1371/journal.pone.0135556 (PMC4535766; doi:10.1371/journal.pone.0135556)
Supplement: S2 Table — In vitro activity profile in IC50 (µM) of all tested compounds. (DOCX) [file pone.0135556.s007.docx]

S2 In vitro activity profile in IC_50_ (μM) of all tested drugs

| **Drug ID** | **^a^T. b. rhod.** | **^b^T. cruzi** | **^c^L. don. axen.** | **^d^L. don. intracell** | **^e^P. falc.** | **^f^Cytotox. L6** | **^g^Cytotox. mac.inf.** | **^h^Cytotox. PMM** |
| --- | --- | --- | --- | --- | --- | --- | --- | --- |
| **Melarsoprol** | **0.01** |  |  |  |  |  |  |  |
| **Benznidazole** |  | **1.99** |  |  |  |  |  |  |
| **Miltefosine** |  |  | **0.44** | **1.21** |  |  |  |  |
| **Chloroquine** |  |  |  |  | **0.13** |  |  |  |
| **Podophyllotoxin** |  |  |  |  |  | **0.017** |  |  |
| Rimantadine | 13.83 | 320.68 | 427.2 | >167.31 | 0.97 | 311.2 | >167.31 | N/A |
| Amantadine | 52.76 | 371.3 | >595.03 | >198.34 | 5.67 | 214.87 | >198.34 | N/A |
| Terbinafine (Hydrochloride) | 95.15 | 40.87 | 23 | 69.54 | 7.72 | 46.97 | >91.49 | N/A |
| Tioconazole | 29.92 | 0.064 | 9.26 | 13.1 | 0.63 | 19.47 | 77.38 | 51.07 |
| Ketoconazole | 33.12 | 0.27 | 38.76 | >18.82 | N/A | 50.99 | 56.45 | N/A |
| Bifonazole | 26.8 | 0.003 | 5.48 | >32.22 | 3.18 | 39.3 | >32.22 | N/A |
| Satranidazole | 18.7 | 19.08 | 10.41 | >103.71 | N/A | >311.13 | >103.71 | N/A |
| Secnidazole | 28.68 | >161.12 | >161.12 | >161.12 | N/A | >483.37 | >161.12 | N/A |
| Ornidazole | 25.93 | 130.08 | >135.97 | >135.97 | N/A | >407.91 | >135.97 | N/A |
| Itraconazole | 8.53 | 0.004 | 2.42 | >1.42 | 1.37 | 1.11 | 4.25 | N/A |
| Clotrimazole | 23.69 | 0.006 | 2.23 | 14.67 | 0.11 | 2.99 | 87 | 32.48 |
| Omeprazole | 51.82 | 68.32 | 90.9 | >86.85 | 13.23 | 125.64 | >86.85 | N/A |
| Albendazole | 14.7 | 12.32 | >113.06 | >3.77 | N/A | 0.41 | 11.31 | N/A |
| Econazole (Nitrate salt) | 41.7 | 0.04 | 6.15 | 13.97 | 0.32 | 15.6 | 67.62 | 22.82 |
| Voriconazole | >286.27 | 11.25 | >286.27 | >85.88 | >143.14 | 194.95 | >85.88 | N/A |
| Miconazole (Nitrate salt) | 36.6 | 0.04 | 4.39 | 10.79 | 0.49 | 15.44 | 62.74 | 23.89 |
| Fluconazole | 273.28 | 9.96 | 210.59 | >97.95 | N/A | >293.85 | >97.95 | N/A |
| Spiperone | 22.35 | 64.58 | >227.57 | >75.86 | 1.76 | 54.36 | >75.86 | N/A |
| Bacitracine | >70.29 | 47.09 | >70.29 | >21.09 | 19.75 | 42.24 | >21.09 | N/A |

S2 continued

| **Drug ID** | **^a^T. b. rhod.** | **^b^T. cruzi** | **^c^L. don. axen.** | **^d^L. don. intracell** | **^e^P. falc.** | **^f^Cytotox. L6** | **^g^Cytotox. mac.inf.** | **^h^Cytotox. PMM** |
| --- | --- | --- | --- | --- | --- | --- | --- | --- |
| Ketotifen | 15.19 | 141 | >290.86 | >96.95 | 0.75 | 147.04 | >96.95 | N/A |
| Paroxetine | 1.13 | 14.36 | >273.25 | >9.11 | 9.23 | 13.84 | 30.36 | N/A |
| Pentamidine | 0.01 | 4.44 | 19.77 | 2.63 | N/A | 8.87 | >29.37 | 7.78 |
| Dapsone | 352.79 | 219.89 | >402.73 | >120.82 | 167.54 | 203.78 | >120.82 | N/A |
| Artesunate | 16.78 | 8.97 | 0.35 | >7.8 | 0.003 | 0.78 | >7.8 | N/A |
| Benfluorex | 21.06 | 73.71 | 243.05 | 54.36 | 6.52 | 49.15 | >85.38 | N/A |
| Auranofin | 0.01 | 2.27 | 0.11 | >1.47 | 1.67 | 4.79 | 4.42 | N/A |
| Cimetidine | >396.28 | 351.11 | >396.28 | >118.89 | 29.17 | 301.57 | >118.89 | N/A |
| Lonidamine | 134.82 | 177.48 | 8.66 | >93.41 | >15.57 | 273.07 | >93.41 | N/A |
| Leflunomide | 212.8 | 152.1 | 21.76 | >111.02 | >18.5 | 123.98 | >111.02 | N/A |
| Lincomycin | >221.38 | >221.38 | >221.38 | >73.79 | 7.08 | >221.38 | >73.79 | N/A |
| Erythromycine (Hydrate) | 72.08 | 113.97 | >132.99 | >39.9 | 9.91 | 92.16 | >39.9 | N/A |
| Nitrofurantoine | 0.5 | 4.35 | 2.12 | >41.81 | N/A | 90.31 | 125.44 | N/A |
| Nifuroxazide | 0.03 | 0.23 | 2.83 | >10.86 | 7.82 | 12.31 | 36.2 | N/A |
| Metronidazole | 337.7 | 278.69 | 491.36 | >175.28 | 276.94 | 338.87 | >175.28 | N/A |
| Nifurtimox | 1.44 | 0.19 | 2.76 | 20.68 | N/A | 87.02 | >34.81 | 15.7 |
| Tinidazole | 16.57 | >80.95 | >80.95 | >80.95 | N/A | >242.86 | >80.95 | N/A |
| Niclosamide | 1.67 | 2.49 | 0.15 | >0.31 | 1.14 | 2.3 | 1.01 | N/A |
| Zidovudine | 139.57 | 155.66 | >374.19 | >112.26 | 174.75 | 206.93 | >112.26 | N/A |
| Stavudine | 119.53 | 340.74 | >446 | >133.8 | 186.43 | 266.26 | >133.8 | N/A |
| Fluoxetine | 2.01 | 19.14 | >290.95 | >9.7 | 1.21 | 14.97 | 32.33 | N/A |
| Mebeverine | 54.24 | 3.89 | >209.52 | 54.94 | 1.74 | 70.77 | >69.84 | N/A |
| Cloperastine | 4.91 | 9.46 | 265.26 | >30.31 | 0.87 | 43.35 | 90.94 | N/A |
| Triamterene | 21.68 | 22.74 | 306.79 | >11.85 | 17.21 | 8.53 | >11.85 | N/A |

S2 continued

| **Drug ID** | **^a^T. b. rhod.** | **^b^T. cruzi** | **^c^L. don. axen.** | **^d^L. don. intracell** | **^e^P. falc.** | **^f^Cytotox. L6** | **^g^Cytotox. mac.inf.** | **^h^Cytotox. PMM** |
| --- | --- | --- | --- | --- | --- | --- | --- | --- |
| Cetirizine (Hydrochloride) | 99.45 | 119.9 | 186.43 | >70.53 | 19.98 | 148.82 | >70.53 | N/A |
| Amphotericin B | 0.76 | 56.69 | 0.34 | 0.31 | 0.8 | 10.27 | 32.4 | 22.39 |
| Indinavir (Sulfate) | 48.19 | 93.55 | >140.89 | >42.27 | 6.03 | >140.89 | >42.27 | N/A |
| Ritonavir | 4.3 | 20.67 | 8.97 | >41.61 | 16.23 | 37.73 | >41.61 | N/A |
| Amprenavir | 28.48 | 105.21 | 112.53 | >59.33 | 15.17 | 159.8 | >59.33 | N/A |
| Tipranavir | 26.38 | 28.21 | 1.64 | >49.78 | 44.63 | 53.43 | >49.78 | N/A |
| Ganciclovir | 251.53 | 240.17 | >391.8 | >117.54 | >195.9 | 179.44 | >117.54 | N/A |
| Atazanavir (Sulfate) | 12.74 | 33.34 | 24.1 | >37.46 | 7.95 | 46.45 | >37.46 | N/A |
| Saquinavir | 12.27 | 17.14 | 77.36 | >44.72 | 11.94 | 18.19 | >44.72 | N/A |
| Darunavir | 31.41 | 92.21 | 125.26 | >54.78 | 46.93 | 155.57 | >54.78 | N/A |
| Lopinavir | 10.81 | 15.38 | 11.83 | >47.71 | 1.92 | 18.92 | >47.71 | N/A |
| Nelfinavir | 20.61 | 10.23 | 9.62 | 20.25 | 10.44 | 12.05 | >52.84 | N/A |
| Famciclovir | 225.31 | 206.95 | >311.2 | >93.36 | >155.6 | 179.87 | >93.36 | N/A |
| Penciclovir | >394.85 | 254.28 | >394.85 | >118.45 | 169.39 | 219.54 | >118.45 | N/A |
| Pyrazinamide | >812.28 | 564.54 | >812.28 | >243.68 | 178.7 | 458.13 | >243.68 | N/A |
| Nicotinamide | >736.94 | >736.94 | >736.94 | >245.65 | >40.94 | >736.94 | >245.65 | N/A |
| Izoniazide | >656.26 | >656.26 | >656.26 | >218.75 | >36.46 | >656.26 | >218.75 | N/A |
| Isoniazide | >729.18 | 382.82 | >729.18 | >218.75 | 246.46 | 602.3 | >218.75 | N/A |
| Ciclopirox olamine | 0.8 | 2.49 | 1.64 | 9.09 | 3.75 | 1.04 | >11.18 | 20.27 |
| Tadalafil | 97.58 | 8.6 | 40.06 | >77.04 | >12.84 | 221.1 | >77.04 | N/A |
| Pyrimethamine | 6.75 | 5.71 | 277.83 | >12.06 | 9.85 | 2.51 | >12.06 | N/A |
| Pirenperone | 29.99 | 128.35 | 161.57 | >76.25 | 4.35 | 232.3 | >76.25 | N/A |
| Dipyridamole | 7.45 | 29.33 | 62.02 | >59.45 | 1.3 | 38.64 | >59.45 | N/A |
| Tafenoquine | 1.42 | 6.11 | 17.91 | 2.16 | 0.27 | 5.52 | 6.47 | N/A |

S2 continued

| **Drug ID** | **^a^T. b. rhod.** | **^b^T. cruzi** | **^c^L. don. axen.** | **^d^L. don. intracell** | **^e^P. falc.** | **^f^Cytotox. L6** | **^g^Cytotox. mac.inf.** | **^h^Cytotox. PMM** |
| --- | --- | --- | --- | --- | --- | --- | --- | --- |
| Mefloquine (Hydrochloride) | 0.53 | 4.48 | 38.58 | 2.41 | 0.0024 | 3.25 | 7.23 | N/A |
| Primaquine | 4.78 | 17.85 | 48.58 | 28.19 | N/A | 43.18 | >38.56 | 39.72 |
| Sitamaquine | 7.39 | 21.98 | 13.65 | 19.48 | 0.08 | 32.31 | >29.11 | 21.37 |
| Chloroquine (Diphosphate) | 3.81 | 99.25 | >196.15 | >58.85 | 0.17 | 50.61 | >58.85 | N/A |
| Ciprofloxacin | 35.91 | 92.05 | >301.8 | >90.54 | 13.19 | 118.01 | >90.54 | N/A |
| Enoxacin | 113.01 | 162.65 | >312.18 | >93.66 | 76.8 | 25.01 | >93.66 | N/A |
| Rifabutin | 12.61 | 27.51 | 56.43 | >35.42 | 1.59 | 60.92 | >35.42 | N/A |
| Rifampicin | 16.74 | >109.36 | 1.53 | >36.45 | 0.1 | 75.22 | >36.45 | N/A |
| Rifaximin | 17.14 | 40.21 | 15.01 | >38.17 | 0.92 | 88.05 | >38.17 | N/A |
| Rifamycin SV (Sodium salt) | 0.99 | 49.95 | 1.5 | >13.87 | 0.55 | 15.68 | 41.62 | N/A |
| Clofazimine | 7.63 | 38.23 | 22.39 | 0.95 | 4.1 | 9.97 | 6.34 | 10.65 |
| Silver sulfadiazine | 18.45 | 24.87 | 125.57 | >12.04 | 1.44 | 20.62 | 40.12 | N/A |
| Griseofulvin | 39.97 | 87.31 | 87.03 | >85.04 | 75.12 | 20.52 | >85.04 | N/A |
| Danazol | 45.93 | 6.25 | 10.16 | >29.63 | 14.13 | 32 | >29.63 | N/A |
| Ganaxolone | 5.47 | 64.96 | 42.46 | >90.22 | >15.04 | 23.49 | >90.22 | N/A |
| Glybenclamide | 87.65 | 93.93 | 9.8 | >60.73 | 38.26 | 116.19 | >60.73 | N/A |
| Doxycycline | 63.68 | 39.15 | 24.08 | >22.5 | 4.48 | 14.49 | 67.5 | N/A |
| Minocycline | 36.55 | 22.3 | 45.68 | >21.86 | 3.58 | 9.42 | >21.86 | N/A |
| Sertraline | 0.77 | 6.76 | 86.4 | >9.8 | 0.51 | 8.1 | 32.65 | N/A |
| Troglitazone | 59.34 | 32.61 | 4.26 | >67.94 | 7.27 | 80.63 | >67.94 | N/A |
| Tolnaftate | 69.29 | 19.97 | 4.33 | 50.1 | 18.99 | 84.58 | >97.59 | N/A |
| Clomiphene | 6.21 | 11.45 | 21.6 | >7.39 | 1.37 | 13.06 | 24.63 | N/A |
| Thioridazine | 0.53 | 5.83 | 22.41 | >7.74 | 1.07 | 5.39 | 25.79 | N/A |
| Triflupromazine | 1.42 | 15.01 | 79.79 | >8.51 | 2.72 | 18.5 | 28.38 | N/A |

S2 continued

| **Drug ID** | **^a^T. b. rhod.** | **^b^T. cruzi** | **^c^L. don. axen.** | **^d^L. don. intracell** | **^e^P. falc.** | **^f^Cytotox. L6** | **^g^Cytotox. mac.inf.** | **^h^Cytotox. PMM** |
| --- | --- | --- | --- | --- | --- | --- | --- | --- |
| Amoxapine | 1.87 | 17.94 | >286.82 | >9.56 | 1.14 | 16.16 | 31.87 | N/A |
| Fluphenazine | 2.03 | 12.98 | 46.69 | >6.86 | 0.5 | 11.54 | 22.86 | N/A |
| Clomipramine | 2.06 | 20.29 | 75.68 | >9.53 | 1.3 | 19.79 | 31.76 | N/A |
| Nortryptyline | 1.17 | 22.89 | >341.71 | >11.39 | 0.58 | 27.87 | 37.97 | N/A |
| Promazine | 2.16 | 49.15 | >316.43 | >35.16 | 0.49 | 30.06 | >35.16 | N/A |
| Chlorpromazine | 1.25 | 17.15 | 48.45 | >9.41 | 3.03 | 12.32 | 31.36 | N/A |
| Amitriptyline | 3.03 | 35.11 | 131.25 | >10.81 | 1.16 | 42.18 | 36.05 | N/A |
| Trifluoperazine | 1.23 | 10.13 | 52.93 | >7.36 | 1.48 | 10.99 | 24.54 | N/A |
| Pizotifen | 3.99 | 30.63 | 215.03 | >33.85 | 1.86 | 45.02 | >101.54 | N/A |

^a^*T. b. rhodesiense* strain STIB 900, trypomastigotes. *^b^T. cruzi*, strain Tulahuen C4, intracellular amastigotes. ^c^*L. don*. axen.: axenic amastigotes of *L. donovani*, strain MHOM-ET-67/L82. ^d^*L. don*. intracell: intracellular amastigotes of *L. donovani* strain MHOM-ET-67/L82. . ^e^*P. falc*.: *P. falciparum* strain K1. ^f^Cytotoxicity on L6 cells. ^g^Cytotoxicity on macrophages infected with *L. donovani*. ^h^Cytotoxicity on peritoneal mouse macrophages

IC_50_ values are means of two independent assays, which varied < ±50%.
